# Seroclearance of Hbsag in Chronic Hepatitis B Virus Patients on Lamivudine Therapy: A 10 Year Experience

**DOI:** 10.5539/gjhs.v7n6p101

**Published:** 2015-04-03

**Authors:** Shahnaz Sali, Muayad A. Merza, Sina Saadat, Nazik H. Mustafa, Farzam Queiky, Davood Yadegarynia

**Affiliations:** 1 Infectious Disease and Tropical Medicine Research Center, Shahid Beheshti University of Medical Sciences, Tehran, Iran; 2 Azadi Teaching Hospital, School of Medicine, Faculty of Medical Sciences, University of Duhok, Duhok, Kurdistan, Iraq; 3 District 1 Mofid High School, Tehran, Iran

**Keywords:** hepatitis B surface antigen, lamivudine, seroclearance, seroconversion

## Abstract

**Introduction:**

The aim of this study was to determine hepatitis B surface antigen (HBsAg) seroclearance rate among patients treated with lamivudine at a specialized tertiary care referral hospital in Tehran, Iran.

**Methods:**

All patients on lamivudine (biovudin®) therapy at a dose of 100 mg/day, who showed seroclearnace between March 2001 and September 2011 were recruited. The main evaluation parameters were duration of HBsAg seroclearance and duration of HBsAg seroconversion. Serum alanine aminotransferase (ALT) and aspartate aminotransferase (AST) levels were evaluated using standard methods. HBsAg seroclearance was defined as two consecutive negative serums HBsAg at least 6 months apart, whereas HBsAg seroconversion was defined as the disappearance of serum HBsAg and the presence of anti-HBs for >6 months.

**Results:**

A total of 203 chronic HBV patients treated with lamivudine at a dose of 100 mg/day were included in the study. HBsAg seroclearance and seroconversion were observed in 11 patients after the initiation of the lamivudine therapy. Overall, in lamivudine responder patients, the mean time to HBsAg seroclearance was 26.90±10.93 months (range: 12-48 months). Furthermore, the responders showed seroconversion after a mean time of 26.90±11.08 months from the initiation of lamivudine therapy. When comparing the characteristics of those who have responded to lamivudine and those who have not responded, baseline HBV-DNA levels was significantly lower in responder than non responder patients (p<0.001). Meantime, there was no difference in age, sex, baseline ALT, AST and liver biopsy score between lamivudine responder and lamivudine non-responder patients.

**Conclusion:**

Despite introduction of tenofovir and entecavir as first line treatment for chronic HBV infection, lamivudine remains to be a low cost, safe and effective drug for HBsAg seroclearnace.

## 1. Introduction

Hepatitis B virus (HBV) infection is a serious public health problem, resulting in approximately 2 billion people have been or currently infected with HBV and there are about 400 million chronic infections worldwide (Hepatitis B Foundation, 2014). It is estimated that about 15-40% of HBV infected people will progress and eventually develop cirrhosis, hepatic failure and hepatocellular carcinoma (HCC) (Lok, 2002). The main way of transmission of HBV is through blood and blood products. In Iran, perinatal transmission and injection drug use are main routes of HBV transmission (Merat et al., 2000). The prevalence of HBV infection in most Middle East countries is considered intermediate to high endemicity. Iran is considered as intermediate hepatitis B surface antigen (HBsAg) positive area with a prevalence rate of 2-7% (Alavian et al., 2007). Diagnosis of HBV infection is made through serological and virological markers. Of these, hepatitis B surface antigen (HBsAg) is the cornerstone for diagnosis of HBV infection. However, other markers are essentially fundamental in differentiating between active and inactive infections. Importantly, hepatitis B e antigen (HBeAg) is indicative of active viral replication (Liaw & Chu, 2009). Meantime, measuring serum alanine aminotransferase (ALT) and HBV-DNA viral load is an important component in evaluating and managing patients with chronic HBV infection (Kennedy et al., 2008). Nowadays, a number of antiviral agents have been approved for the treatment of chronic HBV infection namely interferon therapy, the nucleoside analogues (lamivudine, telbivudine, and entecavir), and the nucleotide analogues (adefovir and tenofovir) (European Association for the Study of the Liver, 2012). The ultimate goal of antiviral therapy for chronic HBV infected patients is seroclearance of HBsAg; however, this can only be achieved in a small proportion of patients (Buster et al., 2008). Seroclearance of HBsAg has been associated with low/undetectable HBV DNA by a sensitive molecular assay, normalization of serum ALT levels, histologic improvement in liver, a decreased risk of HCC, and subsequently prolonged survival (Farci et al., 2004)

Based on HBV infection natural history, the annual incidence of spontaneous HBsAg seroclearance has been estimated to be 1.4% (Kim et al., 2008). However, this rate may be lower in patients living in Middle East countries who typically acquire the infection at birth. Therapy induced HBsAg seroclearance is an unusual finding. Although interferon based therapy has been shown to increase HBsAg seroclearnace above the spontaneous rate of HBsAg loss, HBsAg seroclearance is rare following nucleoside therapy (Chu & Liaw, 2010). Of these, lamivudine therapy is no longer considered to be a first-line therapy for chronic HBV infection; however, it remains effective in suppressing HBV-DNA with ALT normalization and histologic improvement in both HBeAg-positive and HBeAg-negative patients (Liaw et al., 2008). The aim of the study was to determine HBsAg seroclearance rate among patients treated with lamivudine at Labbafinajad Medical Center (LMC).

## 2. Patients and Methods

### 2.1 Setting

The LMC, a specialized tertiary care referral center in Tehran, Iran, is one of the main referring centers of chronic hepatitis B cases.

### 2.2 Ethics Approval

The study was approved by the ethics committee of the LMC.

### 2.3 Study Population

Between March 2001 and September 2011, a total of 203 chronic HBV patients treated with lamivudine (biovudin®) at a dose of 100 mg/day were included in the study. The inclusion criteria were patients with chronic hepatitis who were treated with lamivudine during the study period. The patients were naïve patients, negative tests for hepatitis C virus, hepatitis D virus, human immunodeficiency virus, absence of autoimmune disease. The main evaluation parameters were duration of HBsAg seroclearance and duration of HBsAg seroconversion. Serum alanine aminotransferase and AST levels were evaluated using standard methods (upper limit of normal, 40 IU/L). The viral markers (HBsAg, anti-HBs, HBeAg, and anti-HBe) were measured using the ELISA method (Lok, 2002). Serum HBV DNA level was quantitatively assessed in Keyvan Viral Special laboratory by real Cobas Amplicor and Cobas TaqMan from Roche Diagnostics. The conversion factor for Cobas Amplicor is 5.26 and for Cobas TaqMan is 5.82. The lower detection limit (Cut off) for Amplicor is 200 copies /mL (38 IU/mL) and 30 copies (6 IU/mL) for TaqMan. Patients were selected for treatment according to standard criteria: elevated serum aminotransferase level; HBV viral load more than 10,000 copy/ml (2000 IU/ml) and in some cases based on evidences of necro-inflammatory activity in the liver biopsy samples. One hundred twenty eight out of 203 patients underwent liver biopsy. Liver biopsy was considered when viral load was more than 2000 IU/ml and ALT was 1-2 upper limit of normal. With regard to long term follow up, the patients were screened for HCC with alpha-fetoprotein levels (AFP) and hepatic ultrasound, particularly for high risk patients.

### 2.4 Definitions

Chronic hepatitis B was characterized by the presence of detectable hepatitis B surface antigen (HBsAg) in the blood or serum for longer than six months. Chronic HBV infected patient was defined as having detectable HBsAg, either HBeAg positive or negative, a serum HBV-DNA level >2000 IU/ml, and elevated transaminase level. HBsAg seroclearance was defined as two consecutive negative serums HBsAg at least 6 months apart, which were maintained to the end of the study, normal ALT level, and undetectable serum HBV-DNA level. HBsAg seroconversion was the disappearance of serum HBsAg and the presence of anti-HBs for >6 months. HBeAg seroconversion was defined as two consecutive negative HBeAg and positive HbeAb (Lok, 2002).

### 2.5 Statistical Analysis

Data were collected and analyzed by using SPSS (version 10.0; SPSS Inc, Chicago, IL) software package. Categorical data (responders and non-responders) were compared by X2 test or other variables by the Fisher’s exact test. A p value of less than 0.05 was considered statistically significant.

## 3. Results

### 3.1 Characteristics of Patients

Between March 2001-September 2011, 203 chronic HBV patients on lamivudine therapy were included in the study. The mean age of patients was 43.13±13.40 (range 12-84 years) and 147 (72.41%) were male. Baseline characteristics of the studied population are demonstrated in Table 1.

**Table 1:** Baseline characteristics of patients

Patients characteristics	Study population
Age (yr), mean±SD (range)	43.13±13.40 (12-84)
Gender, male	147 (72.41%)
Gender, female	56 (27.59%)
Presence of family history of HBV infection, n (%)	135 (66.50)
ALT (IU/L), median (range)	109.43±171.15 (10-1424)
AST (IU/L), median (range)	71.87±109.43 (13-780)
HBeAg positive patients	75 (36.95%)
HBeAb positive patients	128 (63.05)
Patients with positive HBV-DNA^1^, n (%)	203 (100%)
Stage (liver biopsy) (range)^2^	1.96±1.42 (0-6)
Grade (Liver biopsy) (range)^2^	5.08±3.26 (0-15)
Score (liver biopsy) (range)^2^	7.05±4.27 (1-21)
1. HBV-DNA level >2000 IU/ml; 2. Only 128 underwent liver biopsy.	

1. HBV-DNA level >2000 IU/ml;

2. Only 128 underwent liver biopsy.

Eleven patients (5.4%) out of 203 chronic HBV patients achieved seroclearnace of HBsAg with lamivudine therapy. The mean age of HBsAg seroclearance patients was 41.45±13.01 (range 25-63 years) and 10 (90.9%) were male and 1 (9.1%) was female. All patients were native Iranian.

### 3.2 Seroclearance and Seroconversion of HBsAg

HBsAg seroclearance and seroconversion were observed in all patients after the initiation of the lamivudine therapy. Table 2 demonstrates time to HBsAg seroclearance and time to seroconversion for each patient.

**Table 2:** Time period of HBsAg loss and appearance of anti-HBs from start of the lamivudine therapy

Duration (month)	Patients										

	1	2	3	4	5	6	7	8	9	10	11
HBsAg loss	18	16	24	20	12	48	40	36	30	24	28

Seroconversion to Anti-HBs	18	18	24	18	12	48	40	38	30	24	26

Overall, the mean time to HBsAg seroclearance was 26.90±10.93 months (range: 12-48 months). Seroconversion occurred after a mean time of 26.90±11.08 months from the initiation of lamivudine therapy. The stratification of the results according to the presence of HBeAg revealed that, in HBeAg negative patients, mean time to HBsAg seroclearance was 23.67±8.39 months and mean time to seroconversion was 22.67±9.00 months. In HBeAg positive patients the intervals were longer with a mean time to HBsAg seroclearance of 31.60±12.68 months and mean time to seroconversion of 32.00±12.08 months.

### 3.3 Comparison Between Responder and Non Responder Patients

The main patient characteristics and values for all parameters studied are shown in Table 3. In total, 137 male and 55 female patients did not achieve HBsAg seroclearance, and 10 male and 1female patients achieved HBsAg seroclearance.

**Table 3:** Comparison of clinical characteristics between responders and non responders of patients with chronic hepatitis B virus on lamivudine therapy

Parameter	Responder (n=11)	Non-responder (n=192)	Odd ratio (95% CI)	P value
Age (Yr)	41.45±13.01	43.23±13.13	-1.7800 (-9.8031-6.2431)	0.662
Sex (M:F)	10:1	137:55	4.015 (0.509-85.817)	0.296
ALT (IU/l)	71.63±48.88	110.65±175.30.13	-39.0200 (143.6979-65.6579)	0.463
AST (IU/l)	56.90±42.31	72.93±112.34	-16.0300 (-83.2242-51.1642)	0.639
Viral load	418497.00±423087.74	3895487.00±345065	-3476990.00 (-3733470.8586-3220509.1414)	0.0001
Stage (liver biopsy)	2.36±1.02	2.00±1.45	0.3600 (-0.5152-1.2352)	0.418
Grade (liver biopsy)	4.63±1.80	5.22±3.33	-0.5400 (-2.5395-1.4595)	0.595
Score (liver biopsy)	7.00±2.68	7.22±4.36	-0.2200 (-2.8438-2.4038)	0.869

HBsAg seroclearance had no statistically significant association with age, sex, ALT, AST and liver biopsy histology results. Patients achieving HBsAg seroclearance had significantly lower baseline ratios of log_10_HBV-DNA (P<0.001), compared with those not achieving HBsAg seroclearance (Table 3). None of the patients in this study developed HCC.

## 5. Discussion

To the best of our knowledge, this is the first report on HBsAg seroclearnace in chronic HBV patients treated with lamivudine therapy in Iran. The goals of treatment in chronic HBV infected patients are to maintain sustained suppression of HBV replication and prevention of relapse of liver disease (Liang, 2009; Sorrell et al., 2009). The disappearance of detectable HBeAg and HBV-DNA is considered an indicator of loss active viral replication (Lok & McMahon, 2009). In general achieving the above goals leads to resolve ongoing hepatocellular damage and reducing the development of cirrhosis and HCC (Doo & Ghany, 2010).

Up to date, there are no effective treatment agents to eradicate HBV from hepatocytes of patients with chronic HBV infection. In this study we investigated lamivudine therapy in inducing HBsAg seroclearance in patients with chronic HBV. Although lamivudine therapy has not been recommended as first line therapy for chronic HBV because of the high resistance rate over time, it is still widely used in many Asian countries including Iran due to low cost, rapid action and proven safety for long term use (Paik et al., 2010; Mahabadi et al., 2013).

In the study population, the patients responded to treatment were generally young. Although our finding was not statistically significant, it was in concordance to other studies that showed loss of HBsAg in lamivudine treated patients at a younger age (Yuen et al., 2007). Therefore lamivudine therapy can result in HBsAg seroclearance at early age group patients. In contrast to lamivudine induced HBsAg seroclearance, spontaneous HBsAg seroclearnce is more common in older age patients (Liaw et al., 1991; Chu & Liaw, 2007). Hence, we believe that HBsAg seroclearance in our study population was attributable to lamivudine treatment. Annually, natural seroclearance of HBsAg occurs approximately at 0.12 to 2.38 in Asian countries and from 0.54-1.98 in Western countries (Chen et al., 2007; Manno et al., 2004; Chu & Liaw, 2010).

Following a long term follow up of the patients, we found that 5.4% of them showed HBsAg seroclearance with lamivudine therapy. Similar finding was documented by Gish et al. (2010); however, the rate of HBsAg seroclearance in that study was lower (2.8%) than our study. Other seroclearance rates have also been reported. In some studies rare HBsAg seroclearance following few years of treatment was reported (Marcellin et al., 2004), whereas in other literature none of the patient treated with lamivudine alone clear HBsAg (Chu & Liaw, 2010). With regards to other anti-HBV drugs, different seroclearance rates are reported in the literatures. Follow up studies showed seroclearance rates of 11% with peginterferon (Marcellin et al., 2006), 8% with tenofovir (Matthews et al., 2008) and 5% with enetcavir (Lai et al., 2006).

The numbers of patients included in this study were limited; however, the study included 5 HBeAg positive and 6 HBeAg negative patients who showed HBsAg seroclearnace on lamivudine therapy alone. Of note, although spontaneous HBsAg seroclearnace could occur in HBeAg positive patients, HBeAg negative patients rarely experience natural seroclearnace (Perrollo, 2009; Kim et al., 2008). Therefore, this study documents potential effect of lamivudine monotherapy in inducing HBsAg seroclearance particularly in HBeAg negative patients. When we compared the characteristics of those who have responded to lamivudine and those who have not responded, baseline HBV-DNA levels was significantly lower in responder than non responder patients (p < 0.001). Hence, the response to therapy in the study population was successful because investigations of the patients did not report high viral load. Therefore, lamivudine therapy can be used as first-line drug only in selected patients with low viral load. Furthermore, although it was not statistically significant, liver biopsy score among responders was lower than non responder patients. It has been documented that lamivudine therapy should be restricted to patients with mild fibrosis and HBV-DNA viral load less than 2 million IU/ml (Mauss et al., 2009). For patients with high level viral level HBV replication (>10^9^ copies/ml) only drugs with a high genetic barrier should be used such as tenofovir and entecavir (Wiegand et al., 2010). In the current study, baseline ALT and AST levels were lower in responder than non responder patients, although we did not find significant statistical association. In contrast to our study, other researchers reported a high baseline aminotransferase level to be associated with higher probability of HBsAg seroclearance (Kim et al., 2014; Chan et al., 2011). Generally, lamivudine has been found to be effective in the normalization of liver enzymes, seroconversion of HBeAg, HBV-DNA suppression and improvement of liver histology (Lai et al., 1998; Dienstag et al., 1999).

Lamivudine induced HBsAg seroclearance was observed in all patients within 48 months from start of the therapy. In HBeAg negative patients, the mean time for HBsAg loss and appearance of anti-HBs was 23.67±8.39 months and 22.67±9.00 months, respectively. Our result was comparable to the study which had done by Marcellin et al. (2006) who showed seroclearance of HBsAg and appearance of anti-HBs at 2 years in HBeAg negative patients treated with peginterferon alpha-2a. Whereas in HBeAg positive patients the time intervals were longer for HBsAg seroclearance and seroconversion. Similarly, Dienstag and his colleagues reported that after a median of 36.6 months of lamivudine therapy, 23% of the patients have lost HBsAg (Dienstag et al., 1999).

In overall, treatment with lamivudine was well tolerated by all patients during the entire observation period. There were no side effects of note among the studied patients. It has been documented that long term lamivudine treatment has an excellent safety profile in patients with chronic HBV infection compensated liver disease (Lok et al., 2003). Furthermore other literatures documented that lamivudine is well tolerated and reduces serum levels of HBV-DNA (Lai et al., 1998; Liang, 2009; Sorrell et al., 2009).

For these patients, it is most likely that the HBV infection has been eradicated since there has been no re-emergence of HBsAg 30 months after cessation of lamivudine therapy and there was no detectable HBV-DNA in the blood. Similar studies on lamivudine therapy found that HBV was eradicated at follow up period of 41 weeks based on no re-appearance of HBsAg and serum HBV-DNA at last follow up (Yuen et al., 2004).

Although the period was 10 years, the study has the typical limitation of small sample size at a single referral center. In this study, we did not check for HBeAg negative viral mutation, which underestimate HBeAg positive patients. Furthermore, the different time periods of diagnosis from one patient to another might be a source of limitation in our study.

In conclusion, in keeping with recorded literatures, despite introduction of tenofovir and entecavir as first line treatment for chronic HBV infection, lamivudine remains to be a low cost, safe and effective drug for HBsAg seroclearnace. This was indicated by the clinical recovery and lifelong immunity from the HBV infection, particularly in patients with low viral load. Lamivudine therapy can result in HBsAg seroclearance at early age group patients, which was observed in HBeAg positive and also in HBeAg negative patients, who seldom experience spontaneous seroconversion.
